# Not Just Transporters: Alternative Functions of ABC Transporters in *Bacillus subtilis* and *Listeria monocytogenes*

**DOI:** 10.3390/microorganisms9010163

**Published:** 2021-01-13

**Authors:** Jeanine Rismondo, Lisa Maria Schulz

**Affiliations:** Department of General Microbiology, GZMB, Georg-August-University Göttingen, Grisebachstr. 8, D-37077 Göttingen, Germany; lisamaria.schulz@uni-goettingen.de

**Keywords:** ABC transporter, antibiotic resistance, Gram-positive bacteria, cell wall

## Abstract

ATP-binding cassette (ABC) transporters are usually involved in the translocation of their cognate substrates, which is driven by ATP hydrolysis. Typically, these transporters are required for the import or export of a wide range of substrates such as sugars, ions and complex organic molecules. ABC exporters can also be involved in the export of toxic compounds such as antibiotics. However, recent studies revealed alternative detoxification mechanisms of ABC transporters. For instance, the ABC transporter BceAB of *Bacillus subtilis* seems to confer resistance to bacitracin via target protection. In addition, several transporters with functions other than substrate export or import have been identified in the past. Here, we provide an overview of recent findings on ABC transporters of the Gram-positive organisms *B. subtilis* and *Listeria monocytogenes* with transport or regulatory functions affecting antibiotic resistance, cell wall biosynthesis, cell division and sporulation.

## 1. Introduction

ATP-binding cassette (ABC) transporters can be found in all kingdoms of life and can be subdivided in three main groups: eukaryotic transporters, bacterial importers and bacterial exporters. In these transporters, the translocation of cognate substrates is driven by ATP hydrolysis [1]. In bacteria, ABC transporters can be involved in the import or export of a wide range of substrates, such as amino acids, ions, sugars or complex organic molecules [2]. For instance, the ABC transporter ZnuABC of *Bacillus subtilis* is required for the uptake of zinc [3], and the transporter TagGH is responsible for the export of wall teichoic acid, a secondary cell wall polymer of Gram-positive bacteria [4,5,6]. ABC exporters can also be involved in the export of toxic compounds, such as antibiotics—for instance, the multidrug transporter Sav1866 of *Staphylococcus aureus* or BmrA of *B. subtilis* [7,8,9].

ABC transporters are typically composed of four core domains: two nucleotide binding domains (NBDs), also referred to as ATP-binding proteins, which hydrolyze ATP, and two transmembrane domains (TMDs), which allow the transport of the substrate across the cell membrane. NBDs and TMDs can be formed by either homodimers or heterodimers [8]. Additionally to NBDs and TMDs, ABC importers possess an extracellular substrate binding protein (SBP), which is required for the capturing and delivery of substrates to the transporter [10]. Energy-coupling factor (ECF) transporters use a membrane-integrated S-component for substrate binding [11].

In past years, it became apparent that ABC transporters are not only involved in the import or export of their cognate substrates. For some ABC transporters it was shown that they use alternative drug detoxification mechanisms or possess regulatory functions, such as FtsEX of *B. subtilis*, which actives the d,l-endopeptidase CwlO via direct protein–protein interaction [12]. In this review, we want to highlight some ABC transporters with alternative detoxification mechanisms or regulatory functions of *B. subtilis* and the human pathogen *Listeria monocytogenes*, which affect antibiotic resistance, cell wall biosynthesis, cell division and sporulation.

## 2. ABC Transporters Involved in Drug Export, Drug Sensing and Detoxification

Antibiotic resistance is a rising problem in modern society, which is associated with enormous costs for the public health sector. Bacteria found different strategies to combat antibiotics: inactivating or degrading the antibiotic itself, changing the antibiotic target, preventing the penetration of the drug into the cell and exporting the drug via efflux pumps. ABC exporters can also be involved in the export of antibiotics; however, different detoxification mechanisms have been proposed in recent years. Here we summarize the findings for the multidrug transporters BmrA and BmrCD of *B. subtilis* and the proposed mechanisms of action for BceAB-BceRS systems of *B. subtilis* and *L. monocytogenes*.

### 2.1. The Multidrug Transporters BmrA and BmrCD

The ABC exporter BmrA of *B. subtilis* is composed of one NBD and one TMD, which are linked by long intracellular domains, and functions as a homodimer [13,14]. Different techniques, such as cryo-electron microscopy, solid state nuclear magnetic resonance (NMR), electron paramagnetic resonance (EPR) spectroscopy and biochemical studies, revealed an inward-facing conformation of apo-BmrA, whereas ATP-bound BmrA is outward-facing [14,15,16,17,18]. Interestingly, the transition from inward to outward-facing conformation only seems to require ATP binding and does not depend on ATP hydrolysis. Whether the release of the substrate of BmrA during translocation is facilitated solely by the switch to the outward-facing conformation or whether this process requires the hydrolysis of ATP is currently unknown [15]. In vitro and in vivo studies showed that BmrA is able to transport several substrates, such as Hoechst 33342, ethidium bromide, doxorubicin and cervimycin C [9,19]. Cervimycin C resistant mutants of *B. subtilis* were isolated, which harbored two mutations in the intergenic region proceeding *bmrA* leading to enhanced *bmrA* transcription and BmrA production. In addition, deletion of *bmrA* leads to a reduction of cervimycin C resistance of *B. subtilis*. Based on these observations, it was suggested that BmrA is involved in cervimycin C resistance in *B. subtilis* [19].

The half transporters BmrC and BmrD consist of one NBD fused to one TMD. Co-purification studies revealed that both proteins form a heterodimer [20]. Alterations in the sequence of the consensus motif of the NBDs result in the presence of one degenerate nucleotide binding site (NBS) and one consensus NBS in the BmrCD complex. In the apo-form, the BmrCD transporter is proposed to have an inward-facing conformation with one ATP bound to the degenerate NBS. Substrate binding to the TMD either occurs before or after the first ATP is bound to the NBS. The binding of a second ATP molecule to the consensus NBS results in a conformational change of the NBDs, which is not transferred to the TMDs. The inward to outward-facing transition of the TMDs is solely driven by the hydrolysis of the ATP molecule bound to the consensus NBS and leads to the release of the substrate [21].

Using inside-out membrane vesicles, Torres et al. could show that BmrCD is able to transport the fluorescent substances Hoechst 33342, doxorubicin and mitoxantrone [20]. BmrCD was also able to translocate ethidium bromide into reconstituted giant unilamellar vesicles, and the transport activity could be inhibited by orthovanadate, an ATPase inhibitor [22]. The genes encoding the BmrCD transporter are induced in the presence of a number of antibiotics, most of which target the ribosomes (e.g., chloramphenicol, erythromycin, gentamycin) [20,23,24]. Surprisingly, deletion of *bmrCD* had no effect on growth in the presence of the tested antibiotics; however, other transporters could also be involved in the detoxification of these drugs and could mask the phenotype [20]. In-depth analysis of the regulation of *bmrCD* expression revealed that it is controlled at two levels: the induction of *bmrCD* is only possible during the transition and stationary growth phase, and the antibiotic-induced expression is regulated by a ribosome-mediated transcriptional attenuation mechanism, which is controlled by the leader peptide BmrB [25]. *bmrB* encodes a small protein of 54 amino acids, which is co-transcribed with *bmrCD* [25]. Upstream of *bmrB* is a binding site for the main transition stage regulator AbrB [26], which is responsible for the growth phase dependent expression of the *bmrBCD* operon [25]. An intrinsic terminator-like structure and alternative anti-terminator and anti-anti-terminator structures are located within the coding region of *bmrB*, which are required for the antibiotic-mediated expression of *bmrCD*. In the absence of antibiotics, the *bmrB* terminator forms, thereby blocking the expression of *bmrCD*. The binding of ribosome-targeting antibiotics such as lincomycin leads to the formation of an anti-termination structure, thereby resulting in the expression of *bmrCD* [25].

The biological role of the growth phase-dependent expression of *bmrCD* is still unknown; however, it was speculated that BmrCD might have a role in sporulation [25]. Indeed, transcriptome data show that *bmrCD* transcript levels are elevated during sporulation [27]. In *B. subtilis*, initiation of sporulation depends on the phosphorylation of the transcription factor Spo0A, which is accomplished by a phosphorelay including the phosphotransferases Spo0F and Spo0B. Spo0F is phosphorylated by several histidine kinases, the major ones of which are KinA and KinB [28,29]. BmrD was identified as an interaction partner of the histidine kinase KinA, and it was speculated that the transporter BmrCD might transfer signals to KinA to initiate sporulation. Furthermore, it was shown that the overexpression of BmrCD reduced sporulation efficiency of a *kinB* deletion strain in a Spo0A-dependent manner, and deletion of *bmrCD* had no effect on sporulation [30]. Further studies are required to identify the exact biological role of BmrCD for sporulation initiation in *B. subtilis*. Homologs of BmrCD can also be found in the non-spore forming Gram-positive bacterium *L. monocytogenes*; however, the functions of Lmo1651 and Lmo1652 have not been investigated so far.

### 2.2. BceAB-BceRS Systems in B. subtilis and L. monocytogenes

In the past few years, several ABC transporters have been identified, which are not only required to inactivate or export drugs, but which are essential for drug sensing. The best-characterized system is the BceAB-BceRS system of *B. subtilis*, which is required for the resistance against bacitracin, actagardine, mersacidin and plectasin [31,32,33,34]. Bacitracin blocks the recycling of lipid carriers during the lipid II cycle of cell wall biosynthesis by binding to the diphosphate lipid carrier undecaprenyl pyrophosphate (UPP), and actagardine, mersacidin and plectasin directly bind lipid II. A phylogenetic analysis revealed that BceAB-like transporters are nearly exclusively found in bacteria of the phylum Firmicutes and confer resistance to a subset of antimicrobials [35,36,37]. These transporters belong to the peptide 7 exporter family, usually consist of two ATP-binding proteins (BceA), a large permease (BceB) and are mostly associated with a BceRS-like two-component system. It has been shown that both components, the transporter BceAB and the two-component system BceRS, are required for sensing of and resistance to bacitracin in *B. subtilis* [35,38,39]. The histidine kinase BceS belongs to the family of intramembrane-sensing histidine kinases whose members lack the extracellular sensing domain [36,40]. *B. subtilis* BceB and other BceB-like permeases possess a large extracellular loop of around 200 to 250 amino acids, which is thought to contain the ligand-binding site [32,34,39]. In the absence of bacitracin, *B. subtilis* produces basal levels of BceAB transporters, which control the conformation of the histidine kinase BceS, thereby keeping BceS in its inactive state [41]. Upon binding of bacitracin, BceAB and BceS form a sensory complex, which detects the activity of BceAB and activates the promoter *P_bceA_* via the response regulator BceR in a dose-dependent manner [33,42,43]. The mechanism by which the BceAB transporter confers resistance to antibiotics, however, has been investigated for nearly two decades and is still highly discussed. In the past, BceAB was thought to function as an efflux transporter [31], but other studies suggested that bacitracin is imported and degraded by BceAB [39]; experimental evidence for both hypotheses is missing. Kingston et al. proposed that BceAB transports UPP across the membrane to the cytoplasmic leaflet, thereby rendering it inaccessible to bacitracin [44]. However, this mechanism would not explain how BceAB confers resistance to the lipid II-targeting antibiotics. A recent study revealed that BceAB specifically recognizes complexes of bacitracin and UPP. The authors further suggest that BceAB breaks this interaction using the energy derived from ATP hydrolysis by BceA, thereby releasing UPP from the inhibitory grip of bacitracin [45]. Thus, BceAB seems to provide resistance to antimicrobials through target protection rather than degradation.

**Figure 1:**
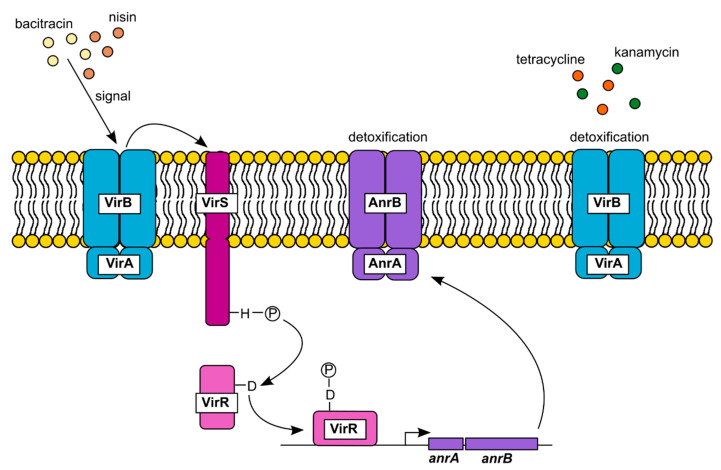
The VirAB-VirRS-AnrAB resistance module of *L. monocytogenes* EGD-e. The ABC transporter VirAB senses the presence of bacitracin, nisin and a number of β-lactam antibiotics. This leads to activation of the intramembrane-sensing histidine kinase VirS, which subsequently phosphorylates the response regulator VirR [46,47]. VirR binds to the VirR consensus sequence present in the promoter region of *anrAB* and induces the expression of both genes [46,51]. The ABC transporter AnrAB detoxifies bacitracin and nisin by a so far unknown mechanism. The detoxification of kanamycin and tetracycline by VirAB occurs in a VirRS-independent manner [46].

The closest homolog of *B. subtilis* BceB in *L. monocytogenes* is AnrB (27% sequence identity), encoded by *lmo2115*. AnrB is part of the ABC transporter AnrAB and forms a multicomponent resistance module together with a second ABC transporter, VirAB, and the two-component system VirRS (Figure 1) [46,47]. This system confers resistance towards nisin, bacitracin, several β-lactam antibiotics, benzalkonium chloride and ethidium bromide [46,47,48,49]. Activation of the histidine kinase VirS depends on the activity of the ABC transporter VirAB; however, it has not been determined yet whether VirAB and VirS form a sensory complex as described for the BceAB-BceRS system of *B. subtilis*, or whether VirAB transports its substrate to a place where VirS can sense it [46,47]. In *L. monocytogenes* strain EGD-e, VirAB is required for sensing of both nisin and bacitracin, whereas sensing of bacitracin seems to be VirAB-independent in *L. monocytogenes* strain 10403S [46,47]. Upon activation, VirS phosphorylates the response regulator VirR, which subsequently induces the expression of the *anrAB* operon. The ABC transporter AnrAB then detoxifies antimicrobials such as nisin and bacitracin [46,48]; however, the exact mechanism by which AnrAB confers resistance is unknown. It is tempting to speculate that AnrAB might also provide resistance via target protection rather than by inactivation or export of the drug. In addition to its role in drug sensing, VirAB is involved in the detoxification of kanamycin and tetracycline in a VirRS-independent manner [46]. Furthermore, it was suggested that Lm.G_1771, the VirB homolog in *L. monocytogenes* serotype 4b strain G, acts as a negative regulator of biofilm formation, potentially by the export of a signal that prevents biofilm formation [50].

In addition to AnrAB, VirR regulates the expression of DltABCD required for d-alanylation of the cell wall polymers teichoic acids and the lysinyl-transferase MprF, which attaches lysine residues onto phosphatidyl-glycerol in the membrane [51,52,53,54]. d-alanylation of teichoic acids and lysinylation of phosphatidyl-glycerol reduce the negative charge of the cell surface, thereby leading to a diminished binding of cationic peptides and human defensins [49,55,56,57]. In addition to its role in conferring resistance towards antimicrobials, the VirAB-VirRS-AnrAB resistance module is also required for virulence of *L. monocytogenes*. Transcriptome studies revealed enhanced expression of *anrAB* during in vitro and in vivo infections [58,59]. Mutants lacking either the histidine kinase VirS or the response regulator VirR produce shorter actin tails during intracellular infection, thereby leading to a reduced ability to spread from cell-to-cell via actin-based motility [47]. Absence of either VirR or VirS also leads to a reduced adherence and entry into human epithelial cells [51]. Taken together, BceAB-BceRS-like systems are involved in drug sensing and detoxification and can have crucial functions for the virulence of pathogenic bacteria.

## 3. ABC Transporters Affecting Cell Wall Biosynthesis and Remodeling

The most prominent target of clinically used antibiotics is the bacterial cell wall. In Gram-positive bacteria, the cell wall consists of a thick layer of peptidoglycan (PG) and secondary cell wall polymers, the so-called teichoic acids. The synthesis of PG starts in the cytoplasm with the production of lipid II, a disaccharide-pentapeptide, which is linked to an undecaprenyl-carrier. In *B. subtilis*, lipid II is then transported across the membrane by the lipid II flippases MurJ and Amj and incorporated into the growing glycan strand by the glycosyltransferase activity of bifunctional penicillin binding proteins or FtsW and RodA. The glycan strands are subsequently crosslinked by the transpeptidase activity of penicillin binding proteins (reviewed in: [60,61]). Several steps of the PG biosynthesis process can be inhibited by antibiotics, for instance, moenomycin blocks the glycosyltransferase activity and β-lactam antibiotics inhibit the transpeptidase activity of penicillin binding proteins [39,62]. During bacterial growth and cell division, PG is constantly remodeled, degraded and recycled [63,64]. These processes depend on the activity of a diverse set of autolytic enzymes, whose activity needs to be tightly controlled and adjusted to the growth stage and environmental growth condition to prevent cell lysis [60,64].

### 3.1. The YtrBCDEF Transporter of B. subtilis

Recent studies suggest that ABC transporters can also have a direct or indirect effect on PG biosynthesis and remodeling. For instance, it has been shown that the overexpression of the YtrBCDEF ABC transporter of *B. subtilis* leads to the production of a thicker PG layer [65]. The transporter YtrBCDEF is encoded in the *ytrGABCDEF* operon and the GntR family repressor YtrA controls its expression [66,67]. YtrA also represses the expression of the *ywoBCD* operon, which codes for a membrane protein of unknown function, a hydrolase and a major facilitator superfamily transporter, respectively [67]. Both operons, *ytrGABCDEF* and *ywoBCD*, are induced in the presence of several cell wall-acting antibiotics, such as ramoplanin, bacitracin and vancomycin [40,67,68]. YtrE was also identified as a marker protein for the inhibition of membrane-bound steps of PG biosynthesis [68]. In addition, it was shown that the expression of the *ytrGABCDEF* operon is also induced after *B. subtilis* was subjected to cold shock [69].

The YtrBCDEF ABC transporter is composed of two nucleotide binding proteins; YtrB and YtrE; two transmembrane domain proteins, YtrC and YtrD and the solute binding protein YtrF. However, it is still unknown whether these components form one or two separate ABC transporters [66,70]. Yoshida et al. suggested that YtrBCDEF might be involved in the import of acetoin, and thus, in the acetoin utilization of *B. subtilis* [66]. *B. subtilis* produces and excretes acetoin during growth in the presence of excess carbohydrates, which is reused during stationary phase and sporulation [71]. The degradation of acetoin depends on the activity of the multicomponent acetoin dehydrogenase enzyme system (AoDH ES) encoded in the *acoABCL* operon [72]. In addition, it has been shown that absence of the acetyltransferase AcuA, which is encoded in the *acuABC* operon and is involved in the inactivation of the acetyl coenzyme A synthetase AscA [73,74], leads to reduced growth and sporulation on acetoin [75]. Utilization of acetoin is also diminished in a *B. subtilis* and *Bacillus licheniformis acuA* mutant [76,77]. How AcuA affects the acetoin catabolism in *B. subtilis* and *B. licheniformis* is still unknown. The utilization of acetoin is also reduced in a *B. subtilis* strain lacking the putative acetoin importer encoded in the *ytrGABCDEF* operon; however, the acetoin catabolism was not completely abolished in this strain, suggesting that *B. subtilis* encodes at least one additional acetoin import system. Interestingly, the expression of the *ytrGABCDEF* operon was not induced in presence of acetoin [66], suggesting that the YtrBCDEF transporter might only have an indirect effect on acetoin utilization. The absence of the transcriptional regulator YtrA further led to a complete loss of genetic competence [65,78]. The loss of competence of the *ytrA* mutant could potentially be explained by an inability of the DNA to reach the competence pilus ComG due to the thicker PG layer produced by this strain [65]; however, further experiments are required to investigate this. In addition, it remains to be elucidated how the function of the ABC transporter YtrBCDEF is linked to PG biosynthesis and acetoin utilization in *B. subtilis*.

### 3.2. The Putative ABC Transporter EslABC of L. monocytogenes

The ATP binding protein EslA and the transmembrane domain protein EslB of *L. monocytogenes* are weak homologs of YtrB and YtrC/YtrD of *B. subtilis* with a sequence identity of 35% and 22%, respectively. *L. monocytogenes* EslA and EslB are encoded in the *eslABCR* operon together with the transmembrane protein EslC and the RpiR regulator EslR [79]. EslA and EslB are predicted to form an ABC transporter; however, it is still unknown whether the transmembrane protein EslC is also part of the ABC transporter or whether it has an independent role of EslAB. Bacterial two-hybrid experiments indicated that EslB and EslC interact with each other [80], and we will thus refer to the transporter as the EslABC transporter. Phenotypic analysis of strains lacking either EslA or EslB revealed a strong increase in lysozyme sensitivity, and deletion of *eslC* does not affect lysozyme resistance of *L. monocytogenes* [80,81,82]. This observation does not rule out that EslC might be part of the ABC transporter; however, it suggests that EslA and EslB have an independent role of EslC. In *L. monocytogenes*, lysozyme resistance mainly depends on the activity of two PG modifying enzymes: the *N*-deacetylase PgdA and the *O*-acetyltransferase OatA. PgdA is required for the deacetylation of GlcNAc residues of the PG backbone, and OatA modifies MurNAc residues with *O*-acetyl groups [83,84]. The PG produced by an *eslB* mutant is more deacetylated and less *O*-acetylated as compared to the PG of the *L. monocytogenes* wildtype strain. Furthermore, deletion of *eslB* results in the production of a thinner PG layer, and thus, the lysozyme sensitivity of the *eslB* mutant is likely caused by both, the decrease in *O*-acetylation and PG layer thickness [80]. The reduction in cell wall thickness in the *eslB* mutant also leads to an increased cell lysis and EslB is thus required for the maintenance of cell wall integrity in *L. monocytogenes* [80]. In addition to its importance for PG biosynthesis, EslB is required for proper cell division in *L. monocytogenes* as cells lacking EslB form elongated cells. Interestingly, localization studies with the early cell division protein ZapA, which is required for FtsZ filament stabilization [85], suggest that these elongated cells form multiple Z-rings [80]. Thus, a process downstream of the recruitment of early cell division proteins, but prior to septum formation seems to be disturbed in absence of EslB. It also remains to be elucidated what the cellular function of the EslABC transporter is. The genes coding for EslABC are co-transcribed in an operon with *eslR* encoding an RpiR transcriptional regulator [79]. RpiR transcriptional regulator are usually involved in the regulation of sugar phosphate metabolic pathways [86,87,88,89,90,91,92], which indicates that EslABC could acts as a sugar importer. However, ABC importers usually require a substrate binding protein for the recognition of their cognate substrates. None of the *esl* genes or genes adjacent to the *eslABCR* operon code for a putative substrate binding protein; thus, it is unlikely that EslABC is involved in the import of sugars. Another possibility is that EslABC is required for the export of certain PG components, which would explain the production of a thinner PG layer by the *eslB* mutant. It is also possible that EslABC has an alternative function, for instance EslABC could be required for the proper localization of other proteins or the regulation of protein activity. Further studies need to be performed to evaluate these hypotheses.

### 3.3. FtsEX of B. subtilis Regulates the d,l-endopeptidase CwlO

**Figure 2:**
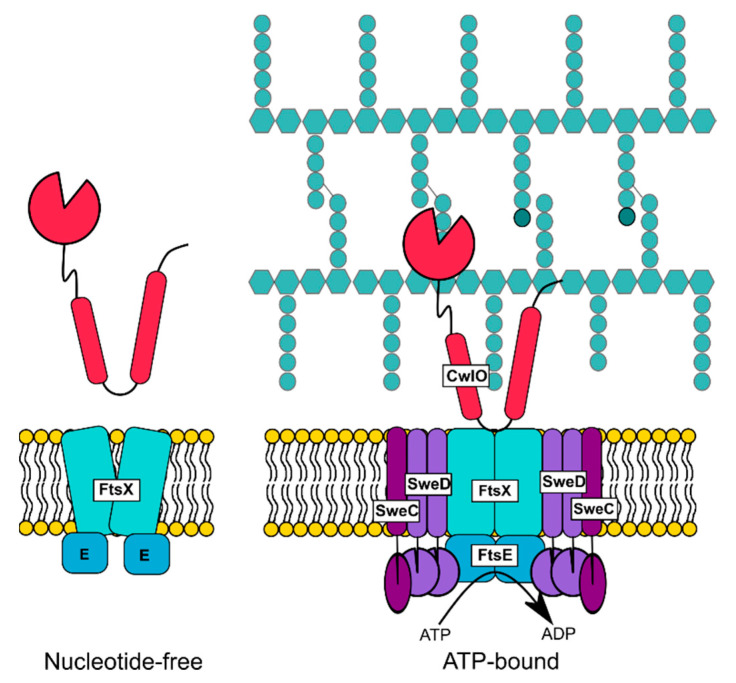
The FtsEX system of *B. subtilis*. The type VII ABC transporter FtsEX is an essential activator of the major cell wall hydrolase CwlO in *B. subtilis*. ATP hydrolysis by FtsE in the cytoplasm is proposed to result in conformational changes of FtsX which allows direct interaction with CwlO and subsequent regulation of its d-l-endopeptidase activity, and the complex is relaxed when in the nucleotide free state. The complex resides in the membrane together with SweC and SweD, that act as essential cofactors (adapted from [99]).

The type VII ABC transporter FtsEX is a transporter involved in PG remodelling. It consists of the transmembrane protein FtsX and the ATP binding protein FtsE and was first identified in the Gram-negative model organism *Escherichia coli*, in which it is essential for cell division as conditional mutants formed filamentous cells (*fts* for filamentous temperature-sensitive) [93]. In contrast to the observation in *E. coli*, initial screens in the Gram-positive bacterium *B subtilis* associated the FtsEX protein complex to polar division and was hence thought to be involved in spore formation in *Bacillus* [94]. The absence of the putative transporter FtsEX in *B. subtilis* led to delayed entry into sporulation and medial septum formation. At the time, it was hypothesized that FtsEX imports a sporulation signal which in turn leads to activation of the master regulator of sporulation, Spo0A. However, later analysis showed that FtsEX stimulates entry into sporulation through its regulation of the autolysin CwlO [12]. CwlO is the major autolysin for cell wall elongation in *B. subtilis*, which hydrolyses the peptide bond between γ-d-glutamate and meso-diaminopimelic acid linkages [95]. Deletion of *cwlO* and *ftsEX* leads to wider and shorter cells with many cells exhibiting a curved or twisted shape, indicating that CwlO and FtsEX share the same pathway [12,96]. Interestingly, the phenotypes associated with *ftsEX* and *cwlO* deletion could be rescued by addition of magnesium to the medium, which is often observed in correlation with disrupted PG biosynthesis. This is in agreement with results of bacterial two hybrid experiments that revealed an interaction between FtsEX and enzymes involved in PG or teichoic acid synthesis and proteins associated with cell elongation [12,96]. An independent study also revealed that absence of CwlO or FtsEX results in decreased competence due to reduced expression of the major regulator of competence, ComK [97]. To further analyse the impact of FtsEX on the hydrolase activity of CwlO, deletions in *ftsEX* or *cwlO* were combined with the deletion of *lytE*, encoding the second major autolysin involved in cell elongation in *B. subtilis*. *lytE* and *cwlO* are synthetic lethal as lack of both autolysins resulted in a disruption of cell elongation and subsequent cell lysis [98]. Synthetic lethality was also observed for the *lytE ftsEX* double mutant. In contrast, a strain lacking FtsEX and CwlO phenocopied the *ftsEX* and *cwlO* single mutants, which again suggests that FtsEX and CwlO are part of the same pathway in *B. subtilis* [12,96]. Deletion of *ftsEX* does not lead to altered CwlO levels and it was thus hypothesized that FtsEX might be involved in the regulation of CwlO hydrolase activity [12]. An in-depth analysis suggests that both, the transmembrane component of the transporter FtsX and the ATP-binding protein FtsE, are required for CwlO functionality. Studies with FtsE variants carrying mutations in residues involved in ATP binding (K41) and hydrolysis (D162) showed that both processes are essential for CwlO activity. In addition, it was shown that CwlO interacts with FtsEX and that FtsX is required for proper localization of CwlO at the cell wall [12]. In addition, SweC and SweD were identified as essential co-factors and binding partners of FtsX, which are required for FtsEX-dependent regulation of CwlO (Figure 2) [99]. Comprehensive analysis of the type VII ABC transporter MacB of *Aggregatibacter actinomycetemcomitans* and *E. coli*, the structural homologue of FtsEX, suggested that it does not seem to transport molecules across the membrane, but rather uses a mechanotransmission mechanism and it was proposed that FtsEX works in a similar manner [100]. Precisely, it was hypothesized, that cytoplasmic ATP hydrolysis via FtsE results in a conformational change of the extracytoplasmic part of the transmembrane protein FtsX, which in turn leads to recruitment and/or activation of CwlO.

## 4. Conclusions

Genomes of Gram-positive and Gram-negative bacteria encode a variety of transport proteins required for the import of molecules such as sugars, amino acids, ions, peptides and for the export of toxic compounds. The classification of transporters into different categories such as ABC transporters or multidrug resistance transporters does not necessarily indicate anything about their function in vivo. Bacteria possess “classical” multidrug resistance ABC exporters, which translocate drugs across the bacterial membrane such as BmrA of *B. subtilis*. ABC transporters can also be involved in the detoxification of drugs by alternative mechanisms—e.g., by target production—as has been described for *B. subtilis* BceAB, or can have an additional impact on the physiology of the organism, as seen for the ABC transporter BmrCD. Some transporters are not even required for the import or export of any substrate; instead, they are involved in the regulation of proteins. The ABC transporter FtsEX is the best-characterized example for such transporters, which directly affects the activity of a PG hydrolase in *B. subtilis* and other Gram-positive and Gram-negative bacteria.

Molecular genetics are helpful to get first insights into the relevance of transporters for the physiology of bacteria; however, an in-depth biochemical analysis is required to fully understand the biological functions of ABC transporters and other transport systems, and to identify their mechanisms of action. These analyses are challenging, as they are often hampered by difficulties in protein preparations required for in vitro assays or for the determination of the 3D structure of the transporters. The genomes of pathogenic and non-pathogenic bacteria harbor numerous transporters, which have not been characterized yet and which might be crucial for the growth and/or lifestyle of these organisms and should thus not be overlooked.
